# KYNURENINE, AN ENDOGENOUS AHR AGONIST, UPREGULATES CXCL12- AND HDAC3-TARGETING MIRNAS INHIBITING OSTEOGENESIS

**DOI:** 10.1093/geroni/igz038.3439

**Published:** 2019-11-08

**Authors:** Ahmed M Elmansi, Nada Eisa, Dmitry Kondrikov, Sadanand Fulzele, Meghan mcGee-Lawrence, Mark Hamrick, Carlos Isales, William D Hill

**Affiliations:** 1 Medical University of South Carolina, Charleston, South Carolina, United States; 2 Department of Orthopaedic Surgery, Medical College of Georgia, Augusta University, Augusta, Georgia, United States; 3 Augusta University, Augusta, Georgia, United States

## Abstract

Osteoporosis is among the most debilitating disorders for aging men and women. Mechanisms underlying loss of bone mass and impaired fracture healing in aged population are not completely understood. Our lab and others reported increased levels of the oxidized tryptophan metabolite, kynurenine (Kyn), in bone marrow mesenchymal stem cells (BMSCs) and interstitial fluid. Here, we report that Kyn significantly inhibits osteogenic differentiation of BMSCs likely via activating aryl hydrocarbon receptor (AhR). KYN significantly downregulated mRNA levels of pro-osteogenic CXCL12 axis, including CXCL12, and its two main receptors CXCR4 and ACKR3. Secreted protein levels of CXCL12 were significantly reduced with KYN, and were rescued upon the use of AhR antagonist 3’,4’-dimethoxyflavone (DMF). Moreover, KYN upregulated levels of pro-aging and CXCL12-targeting miRNAs miR-29b-1-5p and miR-141-3p. Additionally, KYN significantly inhibited mRNA and protein levels of the epigenetic enzyme Hdac3 which is responsible for activating osteogenesis and inhibiting adipogenesis in BMSCs. Using mutagenesis and luciferase assays, we show evidence that miR-141-3p and miR-29b-1-5p directly target CXCL12 3’-UTR. We also show that miR-29b-1-5p directly targets Hdac3 explaining the significantly increased acetylation levels of H4 with KYN treatment. Finally, we show that mRNA levels of both KYN receptor AhR, and KYN-producing enzyme IDO-1 are regulated by CXCL12. We believe this data explains a novel mechanism of the bone aging phenotype and provides multiple potential clinical intervention targets for controlling osteoporosis.

